# High glucose enhances inflammation-driven platelet adhesion to endothelial cells in vitro

**DOI:** 10.1038/s41598-025-28746-4

**Published:** 2025-12-01

**Authors:** Mariangela Scavone, Antonella Fioretti, Martina Molinaro, Claudia Ghali, Carla Martinelli, Tatiana Mencarini, Silvia Bozzi, Nadia Santo, Umberto Gianelli, Monica Miozzo, Marco Guazzi, Gian Marco Podda, Mario Cozzolino, Paola Ciceri

**Affiliations:** 1 Division of General Medicine II, Department of Health Sciences, ASST Santi Paolo e Carlo, University of Milan, Milan, Italy; 2 Laboratory of Experimental Nephrology, Department of Health Sciences, University of Milan, Milan, Italy; 3 Department of Health Sciences, Division of Pathology, University of Milan, ASST Santi Paolo e Carlo, Milan, Italy; 4 Department of Electronics, Information and Bioengineering, Politecnico di Milano, Milan, Italy; 5 Bio-imaging Facility Unitech Nolimits, University of Milan, Milan, Italy; 6 Department of Health Sciences, Division of Medical Genetics, University of Milan, ASST Santi Paolo e Carlo, Milan, Italy; 7 Division of Cardiology, ASST Santi Paolo e Carlo, Milan, Italy

**Keywords:** Endothelial cells, Platelet adhesion, Platelet activation, Diabetes, Inflammation, Glucose, Biological techniques, Lab-on-a-chip, Experimental models of disease, Cardiovascular diseases, Endocrine system and metabolic diseases

## Abstract

Endothelial and platelet dysfunction are central to vascular disease development. We established a simplified, reproducible 96-well plate model to assess platelet adhesion to endothelial cells under conditions mimicking in vitro endothelial dysfunction and platelet hyperactivation. Human aortic endothelial cells (HAEC) were treated with tumour necrosis factor-alpha (TNF-α, 20–50 ng/mL) and/or high glucose levels (30 mM) to replicate in vitro the states of inflammation and hyperglycaemia. Treated HAEC were then exposed to platelets from healthy volunteers. In some experiments, platelets were treated with tirofiban, a GP IIb/IIIa inhibitor, before HAEC exposure. Platelet adhesion was evaluated by fluorescence, transmission, and scanning electron microscopy. Conditions showing significant effects were subsequently confirmed using a microfluidic device under high shear conditions. TNF-α stimulation significantly increased platelet adhesion to HAEC. This was accompanied by morphological changes indicative of activation. High glucose alone had no significant effect but, when combined with TNF-α, it synergistically enhanced platelet adhesion under both static and dynamic flow conditions. Interestingly, adhesion was prevented by platelet pretreatment with tirofiban. This study demonstrates the utility of a straightforward experimental in vitro setup that allows for mechanistic studies regarding platelet-endothelial interaction. By combining simplicity with reproducibility, the model offers a valuable tool for investigating platelet-endothelium interactions. It also serves as a preclinical platform for evaluating therapeutic interventions aimed at reducing thrombotic risk in conditions of inflammation and hyperglycaemia.

**Supplementary Information:**

The online version contains supplementary material available at 10.1038/s41598-025-28746-4.

## Introduction

Platelets are essential for maintaining normal haemostasis and preventing bleeding at the site of vascular injury. Under physiological conditions, circulating platelets do not directly interact with the vessel wall^1^. However, in many disease processes, in response to stimuli, such as endothelial damage, platelet adhesion to the endothelium becomes uncontrolled, leading to thrombus formation. Platelets can also be activated by inflammatory stimuli and in turn may contribute to endothelial dysfunction^2^. Once adhered, platelets can recruit leukocytes, which can further promote endothelial inflammation^3^. Endothelial dysfunction increases the exposure of extracellular matrix (ECM) proteins and upregulates adhesion molecules like P-selectin and E-selectin, which facilitate platelet adhesion. In the presence of damaged or inflamed endothelium, platelet adhesion occurs primarily via interactions with the subendothelium^4^. This process is mediated by a complex interaction between platelet receptors and adhesion proteins, including glycoprotein IIb/IIIa (GP IIb/IIIa). Upon activation, it binds to fibrinogen, fibronectin, and von Willebrand Factor (vWF), mediating a bridging mechanism critical for stable platelet adhesion^4,5^.

Increased platelet reactivity and endothelial dysfunction have been described in several pathological conditions, such as inflammatory diseases and type I and type II diabetes. Both conditions have been associated with an increased risk of thrombotic events^6^. In diabetes, the mechanisms leading to both vascular disease and increased platelet reactivity are closely related to oxidative stress, glucose metabolism, low-grade inflammation, and prothrombotic coagulation abnormalities^7,8^.

From the experimental standpoint, platelet adhesion has been extensively investigated using microfluidic devices^9,10^, which simulate physiological shear stress on endothelial cells and allow for the use of whole blood. Although these systems offer valuable insights, they have several limitations, including technical complexity, limited number of replicates *per* experiment, and short viability of endothelial cells in the microfluidic capillaries. To overcome these challenges and extend the experimental possibilities, we have developed a 96-well plate in vitro model to easily evaluate platelet adhesion to endothelial cells under static conditions. The aim of our study was to establish a model for the simple and rapid evaluation of platelet-endothelium interaction, thus enabling the simultaneous screening of various experimental conditions. We specifically investigated the effect of endothelial inflammation on platelet adhesion and whether high glucose levels affect the inflamed endothelium to exacerbate platelet adhesion, thereby mimicking the endothelial dysfunction observed in type I and type II diabetes.

## Results

### TNF-α treatment of endothelial cells induces platelet adhesion

To establish a model of platelet-endothelial adhesion, HAEC were treated with TNF-α (50 ng/mL) or left untreated for 20 h, followed by exposure to PRP for 30 min at 37 °C. PRP was diluted in either Tyrode’s buffer or autologous platelet-poor plasma (PPP). No significant difference in platelet adhesion was observed between the two diluents in either control or TNF-α treated HAEC (Supplementary Fig. 1 A). In all subsequent experiments we chose to use Tyrode’s buffer due to the potential effects of PPP on platelet function^11^. Different platelet concentrations were tested, showing significantly increased platelet adhesion at the highest platelet concentration of 300 × 10^9^/L in TNF-α- treated HAEC compared to untreated HAEC [% platelet surface coverage: CTRL vs. TNF-α: 0.085 ± 0.020% vs. 0.167 ± 0.063%; at 300 × 10^9^/L; **p < 0.01] (Supplementary Fig. 1B). For subsequent experiments, we selected a concentration of 150 × 10⁹/L, which represents the lower physiological range of normal platelet counts in human blood. This choice was based on its consistency and suitability across donors, as well as minimizing excessive platelet layering, which interferes with washing and quantification. Additionally, we evaluated two TNF-α concentrations, and we found that TNF-α increased platelet adhesion concentration dependently with a significant increase at 50 ng/ml [% platelet surface coverage: CTRL vs. TNF-α: 0.046 ± 0.005%; vs. 0.101 ± 0.011%; **p < 0.01] (Supplementary Fig. 1 C).

**Fig. 1:**
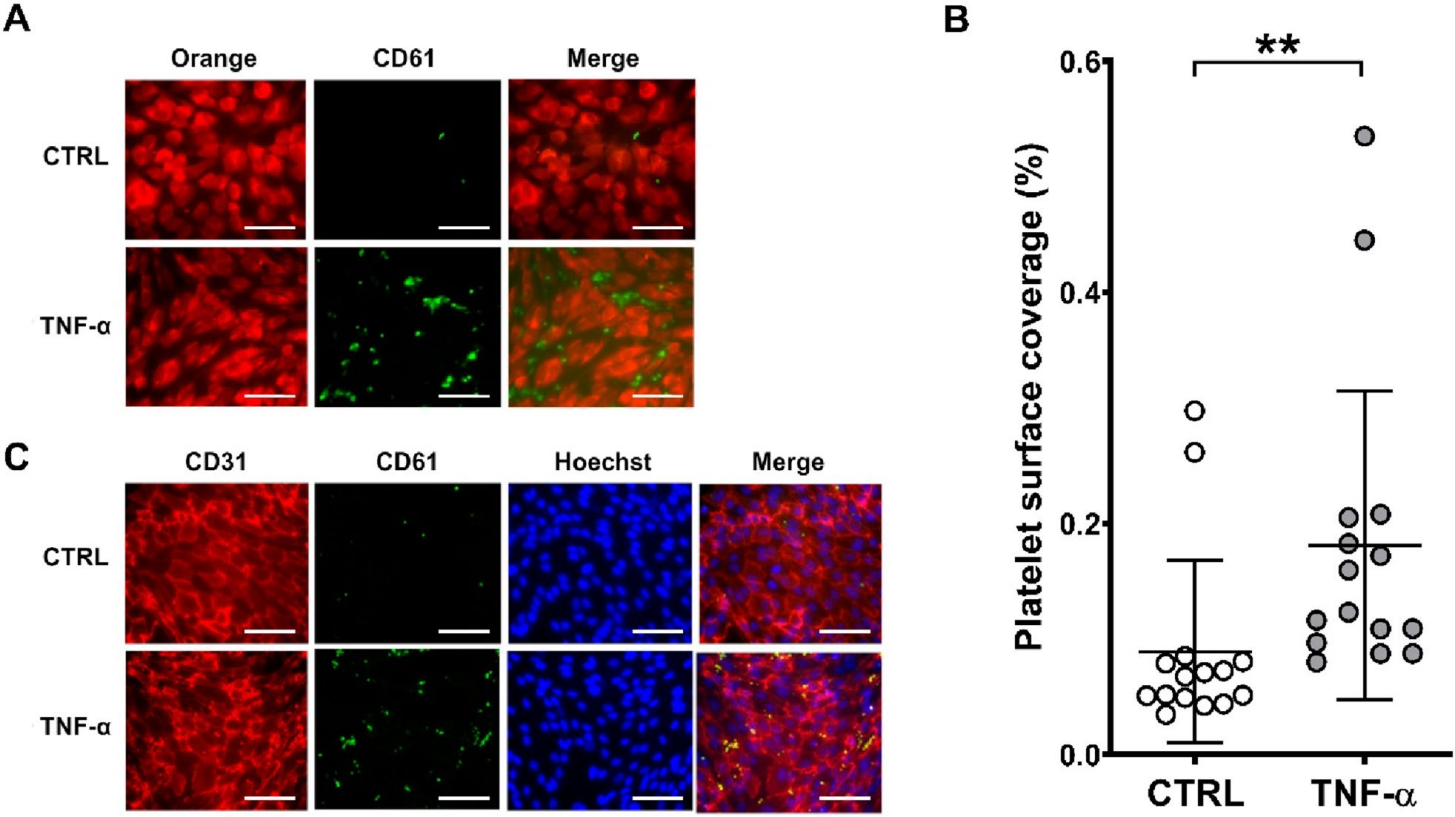
TNF-α treatment of endothelial cells induces platelet adhesion. Platelet adhesion was evaluated on HAEC treated with TNF-α (50 ng/mL) for 20 h compared to untreated controls (CTRL). (**A**) Representative images show Orange tracker-loaded HAEC (red, left), CD61-stained platelets (green, middle) and merged image (right). (**B**) Platelet surface coverage in control and TNF-α treated HAEC is shown. (**C**) Representative images show CD31-stained HAEC (red, left), CD61-stained platelets (green), Hoechst-stained HAEC nuclei (blue) and merged image (right). Scale bars indicate 70 μm. Data are presented as mean ± SD from 15 different experiments. T test (**p<0.01).

Treatment of HAEC with TNF-α (50 ng/mL) induced a significant 2-fold increase in platelet adhesion compared to untreated HAEC [% platelet surface coverage: CTRL vs. TNF-α: 0.089 ± 0.079% vs. 0.181 ± 0.134%; **p < 0.001], although few outliers were observed **(**Fig. 1 A, B). Platelets adhered predominantly along cell margins, as demonstrated by the co-localisation of the endothelial cell membrane marker CD31 (red) and platelet CD61 (green) (Fig. 1 C).

### Glycoprotein IIb/IIIa inhibition reduces platelet adhesion to HAEC

**Fig. 2:**
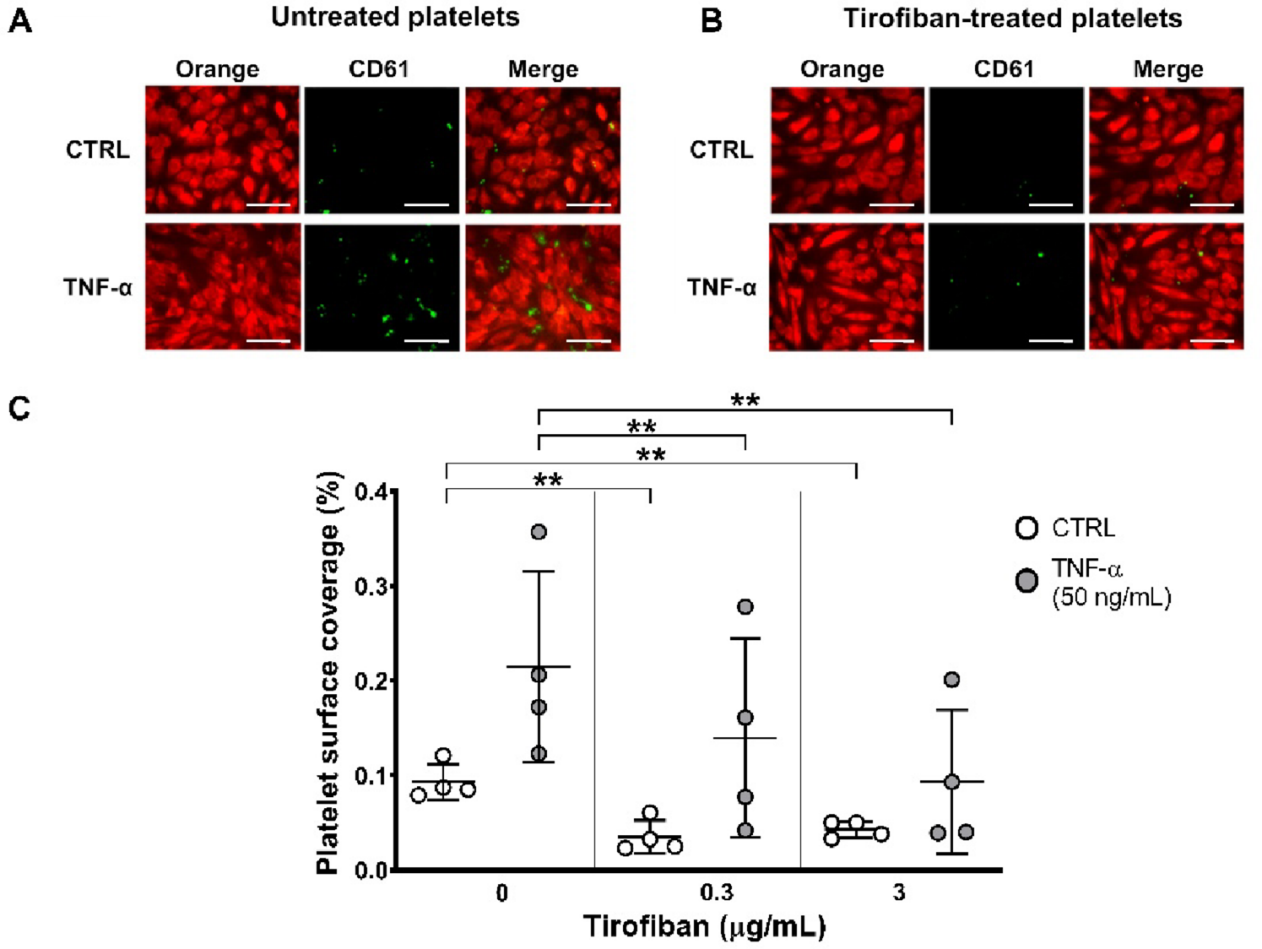
Glycoprotein IIb/IIIa inhibition reduces platelet adhesion to HAEC. Platelet adhesion was evaluated in HAEC treated with and without TNF-α (50 ng/mL), following the pre-treatment of platelets with two different concentrations of tirofiban (0.3 and 3 µg/mL). (**A**) Representative images of untreated and (**B**) tirofiban (3 µg/mL)-treated platelets adhered to Orange tracker-loaded HAEC (red, left), CD61-stained platelets (green, middle) and merged image (right). (**C**) Platelet surface coverage at different concentrations of tirofiban on untreated HAEC and TNF-α-treated HAEC is shown as mean percentage of adherent platelets ± SD of 4 different experiments. Scale bars indicate 70 μm. RM two-way ANOVA followed by Bonferroni’s post hoc test (*p < 0.05, **p < 0.01).

To confirm the role of GP IIb/IIIa in platelet adhesion, platelets were pre-treated with tirofiban, a GP IIb/IIIa inhibitor, before exposure to HAEC. TRAP-induced platelet aggregation was completely inhibited by pre-treatment with tirofiban at two different concentrations (0.3 and 3 µg/mL) [% maximal platelet aggregation: TRAP (20 µM): 0 µg/mL tirofiban = 61 ± 4.5%; 0.3 µg/mL tirofiban = 1 ± 0.4%; 3 µg/mL tirofiban = 0.5 ± 0.3%; **p < 0.01] (Supplementary Fig. 2A-B). In the adhesion assay, tirofiban reduced significantly platelet adhesion to untreated HAEC [% platelet surface coverage: CTRL: 0 µg/mL tirofiban = 0.093 ± 0.019%; 0.3 µg/mL tirofiban = 0.035 ± 0.017%; 3 µg/mL tirofiban = 0.043 ± 0.009%; **p < 0.01] (Fig. 2A, C). In addition, tirofiban reduced significantly platelet adhesion to TNF-α-treated HAEC in a concentration-dependent manner, with maximal inhibition at 3 µg/mL [% platelet surface coverage: TNF-α: 0 µg/mL tirofiban = 0.215 ± 0.101%; 0.3 µg/mL tirofiban = 0.140 ± 0.105%; 3 µg/mL tirofiban = 0.093 ± 0.076%; **p < 0.01] (Fig. 2B, C).

### Adherent platelets on TNF-α treated HAEC exhibit morphological signs of activation

**Fig. 3:**
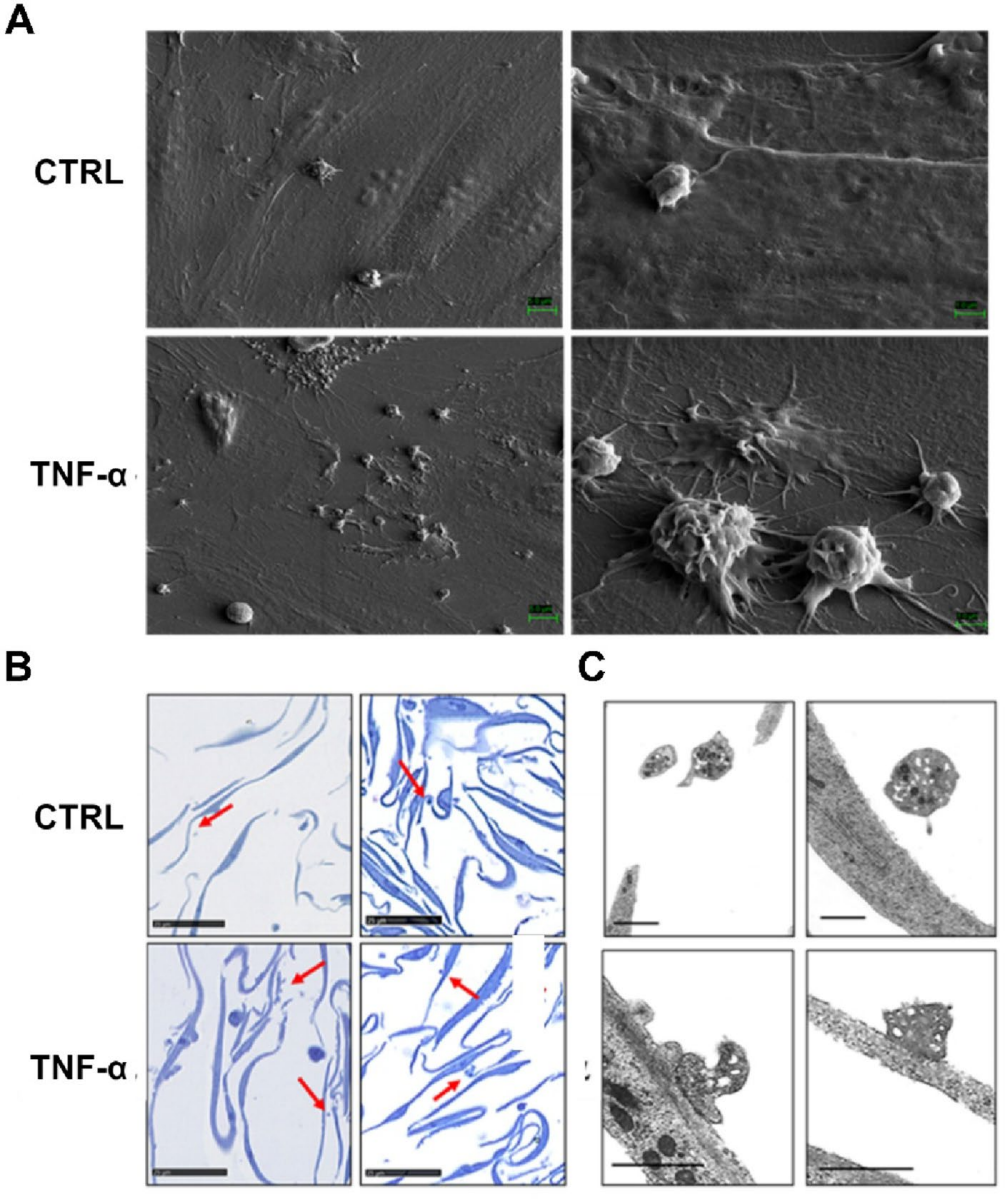
Adherent platelets on TNF-α-treated HAEC exhibit morphological signs of activation. Representative images of platelet adhesion to untreated and TNF-α-treated HAEC. (**A**) SEM images showing platelet adhesion. Scale bars indicate 5 μm for the left images and 1 μm for the right images. (**B**) Light microscopy images of semi-thin sections, with red arrows indicating platelets adhered to HAEC. Scale bars indicate 25 μm. (**C**) TEM images showing ultrastructural details of adherent platelets. Scale bars indicate 1 μm.

To illustrate representative examples of platelet morphology, SEM images were used to visualize the three-dimensional morphology of adherent platelets. On untreated HAEC few and non-activated platelets were observed. In contrast, platelets adhered to TNF-α-treated HAEC exhibited classic signs of activation, including elongated pseudopods, rougher surface, and extensive spreading over the cells, resulting in a flatter appearance (Fig. 3A).

TEM imaging further showed that platelets on TNF-α-treated HAEC tended to cluster along cell extensions, as observed in ultrathin sections (Fig. 3B). Ultrastructural analysis of untreated HAEC showed few adherent platelets, characterised by a more spherical shape and visible storage granules (alpha and dense granules) in the cytoplasm (Fig. 3C). These platelets were mostly close to the endothelial cells but not adherent. In contrast, in TNF-α-treated HAEC, the morphology of adherent platelets changed from discoidal or spherical to an irregular shape, along with a reduction in internal granules, indicating granule release and thus platelet activation (Fig. 3C).

### Platelet activation with TRAP does not affect adhesion to TNF-α-treated HAEC

To assess how platelet activation affects platelet adhesion to endothelial cells, platelets were activated with TRAP at both sub-aggregating 5 µM and aggregating 20 µM concentrations (data not shown).

TRAP stimulation at 20 µM resulted in a slight increase in platelet adhesion, both to untreated and to TNF-α treated HAEC although these changes were not statistically significant, suggesting that TNF-α may already predispose the endothelial cells to maximise platelet adhesion, making further platelet TRAP stimulation irrelevant **(**Supplementary Fig. 3).

### High glucose and TNF-α co-treatment increases platelet adhesion under static conditions

**Fig. 4:**
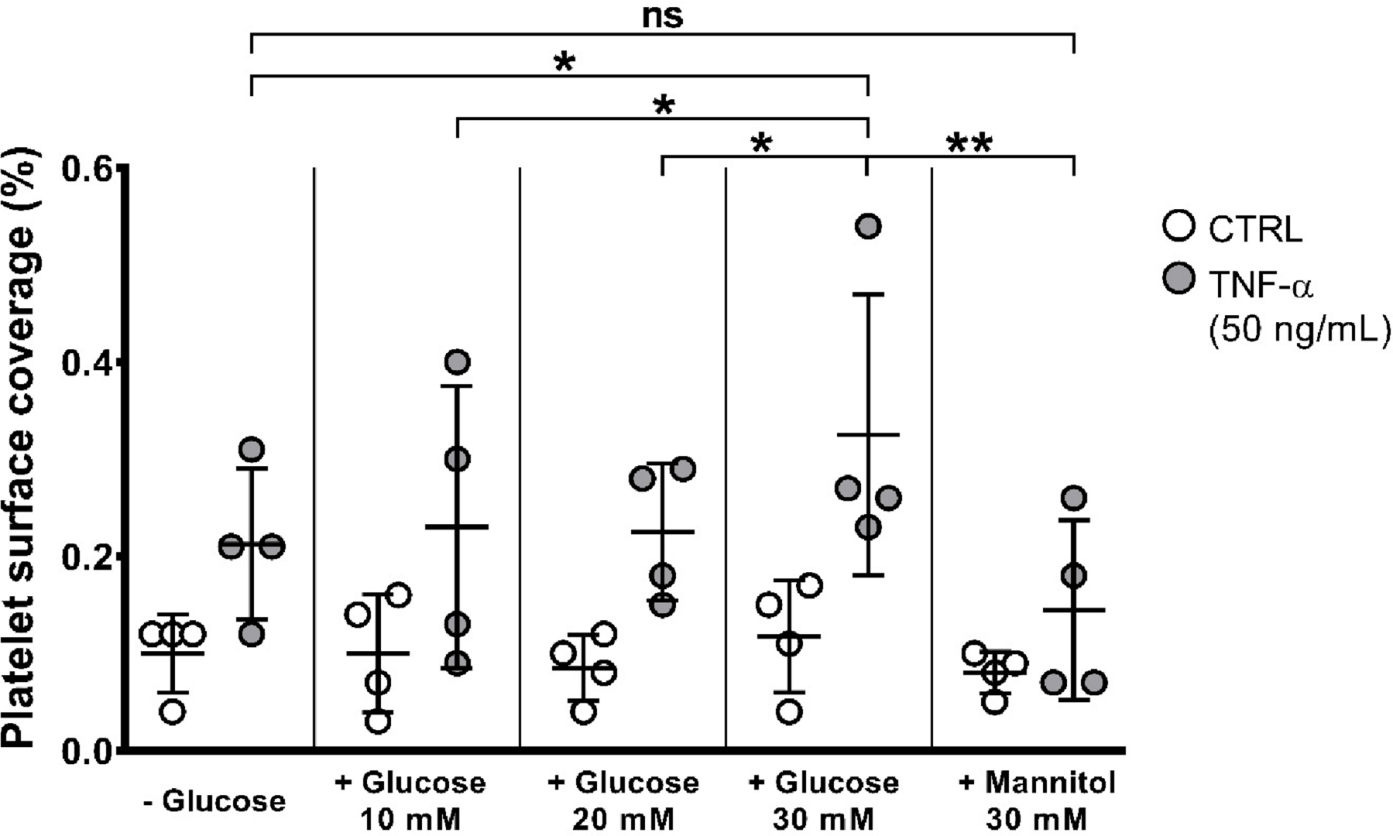
Concentration-response curve of high glucose effect on platelet adhesion to HAEC. HAEC were treated for 20 h with increasing concentrations of glucose (0, 10, 20 and 30 mM) or with 30 mM mannitol, followed by another 20 h of co-treatment of glucose and TNF-α, prior to the adhesion assay. A significant increase in platelet adhesion was observed only at 30 mM glucose, whereas mannitol did not induce any increase. Data are shown as the mean percentage of adhering platelets ± SD from 4 different experiments. RM two-way ANOVA followed by Bonferroni’s post hoc test (*p < 0.05, **p < 0.01).

We investigated how high glucose treatment on both untreated and TNF-α-treated HAEC influences platelet adhesion. To determine the optimal glucose concentration, we tested increasing concentrations of glucose (10, 20 and 30 mM), as well as 30 mM mannitol, as osmotic control. Glucose did not induce platelet adhesion in control HAEC as indeed 30 mM mannitol both on control and TNF-α treated cells (Fig. 4). Conversely, glucose caused a significant increase in TNF-α induced platelet adhesion but only at 30 mM, whereas lower glucose concentrations were not effective [% platelet surface coverage: TNF-α: -glucose vs. + 30 mM glucose; 0.213 ± 0.078% vs. 0.325 ± 0.144%; *p < 0.05, **p < 0.01)] (Fig. 4). Based on these results, 30 mM glucose was used in all subsequent experiments.

**Fig. 5:**
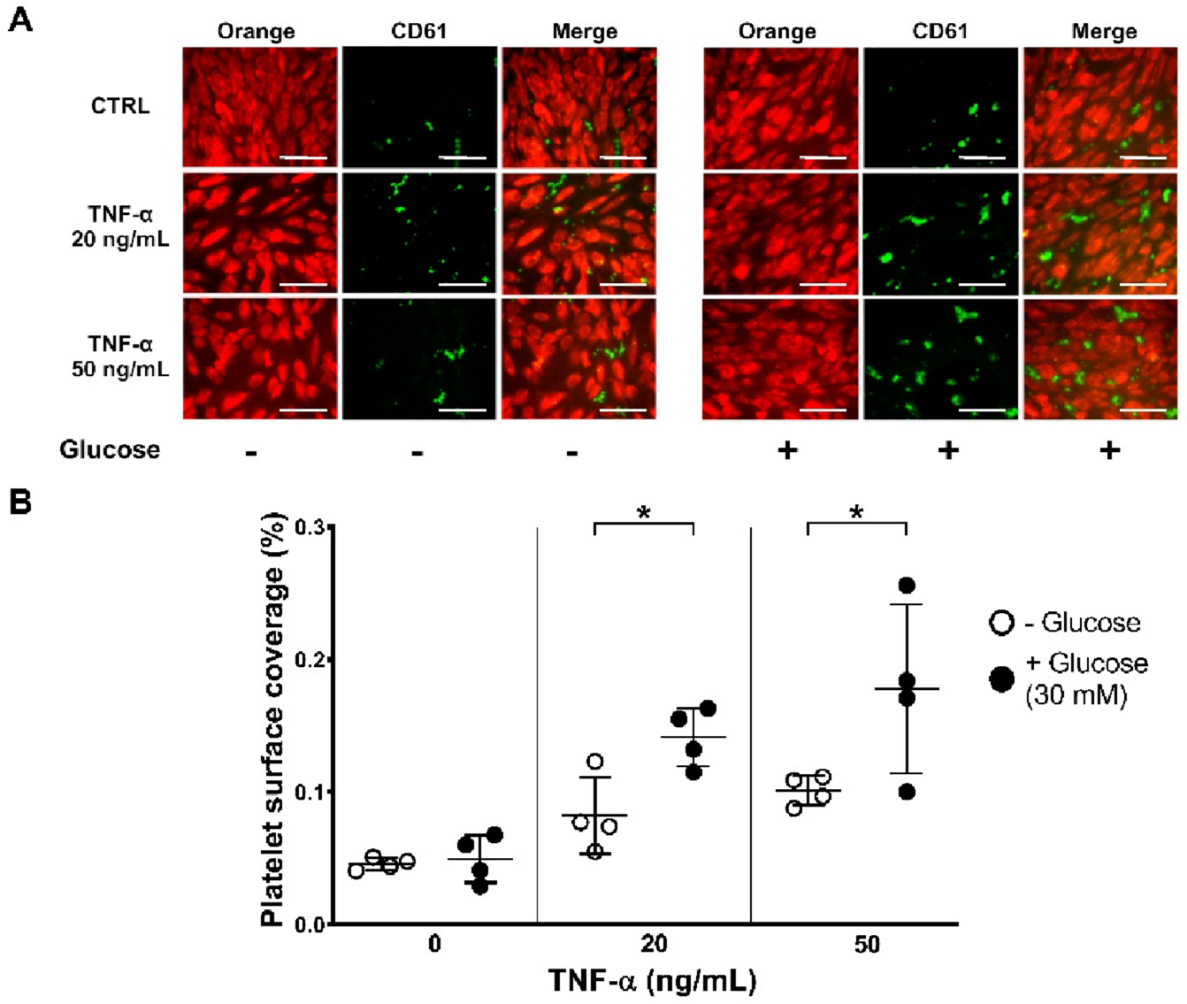
High glucose and TNF-α co-treatment exacerbates platelet adhesion in static conditions. Platelet adhesion was evaluated in HAEC treated with TNF-α (20 or 50 ng/mL) for 20 h and co-treated with or without glucose (30 mM) for total 40 h. (**A**) Representative images show Orange tracker-loaded HAEC (red, left), CD61-stained platelets (green, middle) and merged image (right). (**B**) Platelet surface coverage increases on TNF-α-treated HAEC compared to controls and glucose further increases TNF-α-induced platelet adhesion. Data are shown as mean percentage of adhering platelets ± SD from 4 different experiments. Scale bars indicate 70 μm. RM two-way ANOVA followed by Bonferroni’s post hoc test (*p < 0.05, **p < 0.01).

We then tested the effect of 30 mM glucose on a sub-effective (20 ng/ml) and an effective (50 ng/ml) TNF-α concentration finding that high glucose significantly increased platelet adhesion [% platelet surface coverage: -Glucose vs. + Glucose: 20 ng/mL TNF-α: 0.082 ± 0.029% vs. 0.141 ± 0.022%; 50 ng/mL TNF-α: 0.101 ± 0.011% vs. 0.178 ± 0.064%; *p < 0.05] (Fig. 5A, B). The fold increase in platelet adhesion in TNF-α-treated HAEC compared to untreated HAEC was 1.7 for both TNF-α concentrations, 20 and 50 ng/ml.

### High glucose and TNF-α co-treatment enhances platelet adhesion under high shear conditions

**Fig. 6:**
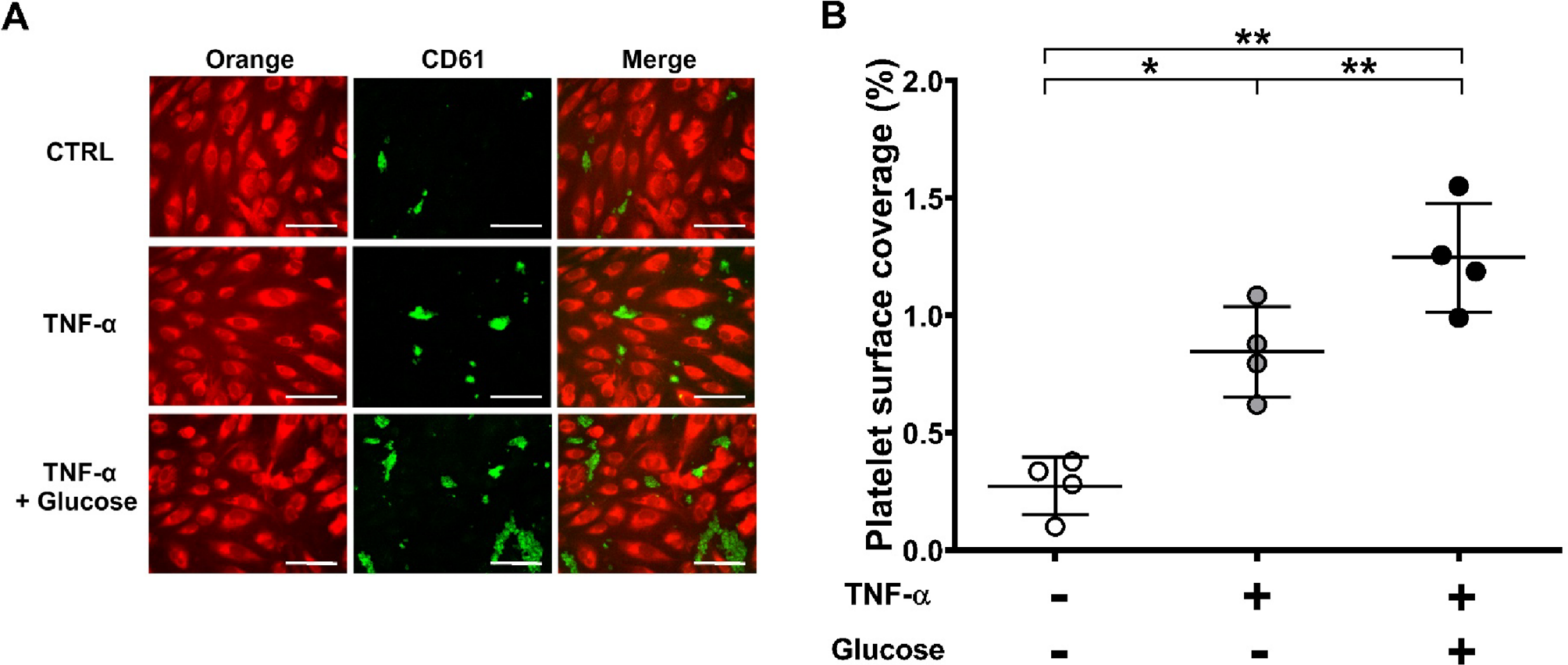
High glucose and TNF-α co-treatment exacerbates platelet adhesion under high shear rate. Platelet adhesion was evaluated using a flow device and perfusing whole blood anticoagulated with hirudin through HAEC-coated channels. (**A**) Representative images show Orange tracker-loaded HAEC (red, left), CD61-stained platelets (green, middle) and merged image (right). (**B**) Platelet surface coverage increases in TNF-α-treated HAEC channels compared to control channels and glucose further increases TNF-α-induced platelet adhesion. Data are shown as mean percentage of adhering platelets ± SD from 4 different experiments. Scale bars indicate 70 μm. RM one-way ANOVA followed by Tukey’s post hoc test (*p < 0.05, **p < 0.01).

**Fig. 7:**
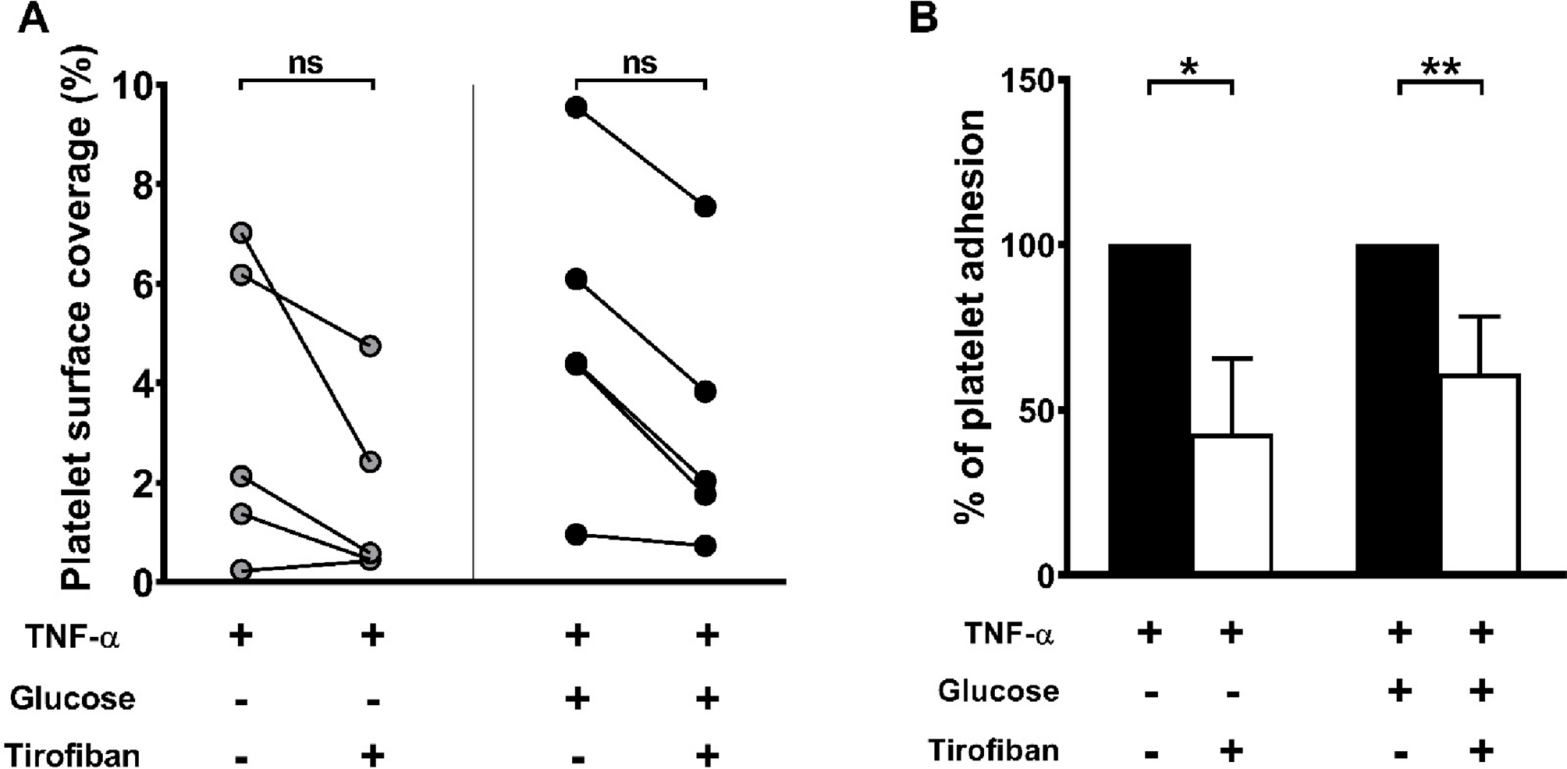
GP IIb/IIIa inhibition with tirofiban reduces platelet adhesion under high shear conditions. Platelet adhesion was assessed under flow (950/s) using a microfluidic system with hirudin-anticoagulated whole blood perfused through HAEC-coated channels. (**A**) Pre-treatment with tirofiban (3 µg/mL) showed a trend towards a reduction in platelet adhesion in both TNF-α-treated and TNF-α + glucose co-treated HAEC under flow conditions,, although this reduction was not statistically significant. (**B**) Defining as 100% platelet adhesion in TNF-α and TNF-α + glucose, platelet adhesion significantly decreases when blood is treated with 3 µg/mL tirofiban. Data are presented as before-after graph and analysed using a RM one-way ANOVA (**A**), or % of platelet adhesion and analysed using a one sample t test (**B**) from 5 independent experiments (*p < 0.05, **p < 0.01).

To confirm the findings from the static adhesion model, we performed platelet adhesion assays under high shear rate conditions (950/sec) using a microfluidic system. This shear rate was chosen to mimic pathological flow conditions, such as those occurring in stenotic vessels^12,13^. Channels coated with HAEC treated with TNF-α (40 ng/mL) alone or in combination with high glucose (30 mM) showed a significant increase in platelet adhesion compared to untreated HAEC [% platelet surface coverage: CTRL = 0.274 ± 0.122%; TNF-α = 0.845 ± 0.193%; TNF-α + Glucose = 1.246 ± 0.232%; *p < 0.05, **p < 0.01] **(**Fig. 6A, B). Moreover, blood pre-treatment with the GP IIb/IIIa inhibitor tirofiban (3 µg/mL) showed a trend towards reduction in platelet adhesion to HAEC treated with TNF-α alone or with TNF-α + glucose under flow conditions (Fig. 7A). Calculating the inhibition of platelet adhesion after pre-treatment with tirofiban as percentage of adhesion, assuming adhesion in the TNF-α treated HAEC of 100%, showed a significant reduction of −57% and − 39% in absence and presence of glucose and TNF-α co-treatment, respectively (Fig. 7B).

## Discussion

In the present study, we have developed and validated a simple and reproducible, 96-well plate in vitro model to study platelet adhesion to endothelial cells under static conditions. This model enables the investigation of platelet-endothelium interactions under different conditions, such as high glucose concentrations or inflammatory stimuli. The key strength of this model lies in its simplicity and adaptability, making it an excellent tool for preliminary studies before more complex and resource-intensive microfluidic experiments (Supplementary Fig. 4). The value of this in vitro plate-based assay is the possibility to test different treatments and drugs in replicate within the same experiment using platelets from the same donor. This allows for comparison while minimizing inter-experiment variability. For example, we could perform the concentration-response experiments to assess the effect of glucose on platelet adhesion using this plate based static assay. This approach would provide a simple and affordable way to have preliminary data, which are essential for identifying the most relevant conditions to be subsequently investigated under flow in more physiologically relevant microfluidic experiments. Reproducibility and meaningful results can be ensured by adapting the conditions optimised in the static model for dynamic assays. Despite its simplicity, the model effectively replicates critical aspects of platelet-endothelial interactions and provides important insights into the mechanisms driving these interactions.

Our results clearly show that inflammation, induced by TNF-α treatment of HAEC, significantly enhances platelet adhesion to endothelial cells under both static (96-well plate) and flow conditions. Although the absolute differences in platelet surface coverage may appear modest and, as expected, platelet adhesion does not result in a measurable reduction in platelet count, these effects could be amplified in vivo, potentially promoting thrombus formation and vascular inflammation. This finding is consistent with established research showing that endothelial inflammation contributes to an increased risk of thrombotic events by enhancing platelet-endothelial interactions^14–17^. The model’s ability to replicate these well-documented effects highlights its relevance and reliability for investigating thrombotic mechanisms under different conditions. Additionally, the model’s flexibility allows for advanced imaging techniques, such as TEM and SEM,, providing detailed insights into platelet morphology and adhesion patterns. In fact, performing experiments under static conditions allows for choosing the appropriate culture support to harvest cells for TEM processing or for growing cells in plastic supports suitable to fit on SEM stub. For instance, ultrathin sections demonstrate that platelets adhere to the peripheral part of the endothelial cells, thus confirming the specificity of our experimental setting. This finding, therefore, dispels any doubt about any non-specific platelet binding to the underlying plastic in a hypothetical space between the cells. Moreover, TEM imaging showed that platelet adhesion to TNF-α-treated HAEC is characterized by significant morphological changes and degranulation, indicative of platelet activation^18^. SEM imaging further confirmed these findings, showing pseudopod extension and preferential platelet adhesion near cell junctions. This observation is crucial, as it highlights the specificity of platelet adhesion to inflamed endothelial cells, even in areas where traditional fluorescent staining may fail to detect the endothelial structure due to the cells’ thinness. Indeed, CD31 staining confirmed that platelets adhere directly to the endothelial membrane, thus validating the model’s ability to accurately mimic in vivo conditions.

Our study also highlights the ability of the model to assess the role of specific platelet receptors in mediating adhesion. Inhibition of GP IIb/IIIa with tirofiban^19^ significantly reduced platelet adhesion under static conditions (96-well plates) and showed a trend towards a reduction under flow conditions. This confirms the critical role of this receptor in the adhesion process^20^. These results validate the functionality of our model and its potential for the preclinical evaluation of anti-thrombotic therapies. The model provides a controlled and reproducible environment, providing a strong basis for subsequent in vivo studies and drug development.

Additionally, our model allowed us to distinguish between the effect of platelet activation and endothelial inflammation on platelet adhesion. Although not statistically significant, TRAP-induced platelet activation was associated with a trend toward increased adhesion to untreated endothelial cells. However, when endothelial cells were pre-treated with TNF-α, further activation of platelets by TRAP did not increase adhesion, suggesting that endothelial inflammation plays a more dominant role in promoting platelet adhesion, at least in vitro. This observation is consistent with previous studies, further confirming that endothelial inflammation is a critical determinant of platelet adhesion under pathological conditions^21^.

One of the most intriguing findings of our study is the synergistic effect observed when high glucose and TNF-α were combined under both static (96-well plates) and flow conditions. While high glucose alone did not significantly affect platelet adhesion, its combination with TNF-α led to a marked increase, suggesting that hyperglycemia amplifies the pro-thrombotic environment caused by endothelial inflammation. This result is particularly relevant in diabetes, both type I and II, where chronic inflammation and hyperglycemia together raise the risk of thrombosis^22^. These findings suggest that hyperglycemia, in combination with inflammation enhances the pro-adhesive properties of endothelial cells, likely through mechanisms involving endothelial dysfunction and oxidative stress. Hyperglycemia is known to promote endothelial dysfunction, increase oxidative stress, elevate reactive oxygen species production (ROS), and reduce nitric oxide (NO) synthesis^23^.

The broader implications of our findings are significant for understanding platelet-endothelial interactions under several conditions. The observed exacerbation of platelet adhesion in the presence of both inflammatory and hyperglycemic stimuli suggests that therapeutic strategies aimed at concurrently controlling inflammation and blood glucose levels could be essential in reducing thrombotic risks in patients with diabetes, both type I and II, and other inflammatory disorders. The model developed here is well-positioned to facilitate further exploration of the molecular pathways involved in these synergistic effects, potentially leading to the identification of novel therapeutic targets.

Study limitations. The model developed in this study offers significant advantages, but there are also limitations that need to be taken into account. The static nature of the initial adhesion experiments does not fully replicate the dynamic conditions of blood flow in vivo, which are crucial for understanding the real-time interactions between platelets and endothelial cells. Although the inclusion of high shear rate conditions in later experiments mitigates this limitation, the model still lacks the complexity of fluid dynamics seen in microfluidic systems. Additionally, this model utilized high TNF-α and glucose concentrations that do not reflect in vivo levels under physiological or pathological conditions but allowed the recreation of in vitro conditions suitable for observing platelet-endothelium interactions over a short period. Furthermore, these conditions may not adequately reflect the prolonged effects of chronic exposure to inflammatory or hyperglycemic conditions. Future studies should aim to integrate long-term culture systems and explore additional factors that influence platelet adhesion, such as other inflammatory cytokines and varying glucose levels over time.

In conclusion, the in vitro model developed and validated in this study offers a powerful and versatile tool for investigating platelet-endothelial interactions under various experimental conditions. By providing a simpler yet highly effective alternative to more complex systems, this model not only enhances our understanding of the mechanisms underlying thrombosis, but also serves as a critical platform for the development and testing of interventions aimed at mitigating thrombotic risks in diseases characterized by platelet hyperactivation, inflammation and metabolic dysfunction. Our work underscores the importance of robust and well-validated models in advancing both basic research and therapeutic development in the context of cardiovascular disease.

## Materials and methods

### Enrolment of healthy volunteers

Healthy volunteers [*n* = 26, 9 men, age range: 32 years (28–43)] were recruited in the morning, from the staff of the ASST Santi Paolo e Carlo and the Università degli Studi di Milano. All participants were allowed to consume a light breakfast at home before blood collection and abstained from medications known to affect platelet function for at least 10 days prior to enrolment. The study was approved by the institutional ethics committee (Comitato Etico ASST Santi Paolo e Carlo) and conducted in accordance with the principles of the Helsinki Declaration. All subjects gave their written informed consent to participate to the study.

### Blood collection

Venous blood samples were collected from an antecubital vein through a 21-gauge butterfly needle, without a tourniquet to minimize platelet activation. The first 4 mL of blood was collected in K2E EDTA tubes (Greiner, Milano, Italy) for complete blood cell count using a Coulter haematology analyser (Medonic M series 16, Milan, Italy). Additional blood samples were anticoagulated with hirudin (S-Monovette 1.6 mL Hirudin; SARSTEDT, Nümbrecht, Germany), gently mixed, and allowed to rest at room temperature (RT) for 15 min before use. The hirudin anticoagulated blood was then centrifuged at 200 *x g* for 10 min to obtain platelet-rich plasma (PRP). All experiments were conducted using platelets from independent healthy donors; each condition was tested in at least three different donors, and each measurement was performed in duplicate for each donor.

### Measurement of platelet reactivity to exogenous agonist by light transmission aggregometry

In each experiment, to verify platelet reactivity in healthy volunteers, platelet aggregation was assessed by light transmission aggregometry (LTA) using the ALAT-2 analyser (BIOLA Ltd., Moscow, Russian Federation). PRP was incubated in aggregation cuvettes at 37 °C with Thrombin Receptor Activating Peptide (TRAP, 5 and 20 µM; Sigma-Aldrich, St Louis, MO, USA). Changes in light transmission were recorded for 6 min. Platelet aggregation was also assessed after stimulation with TRAP (20 µM) in the presence of a glycoprotein IIb/IIIa inhibitor (tirofiban, 0.3 and 3 µg/mL; Aggrastat, Correvio Pharma, Vancouver, Canada) to verify platelet inhibition.

### HAEC culture

Human Aortic Endothelial Cells (HAEC) were purchased from ATCC and cultured on 0.1% w/v gelatin-coated plates in growth medium (Vascular Cell Basal Medium containing 100 U/mL penicillin, 0.1 mg/mL streptomycin and supplemented with Endothelial Cell Growth Kit-BBE). HAEC were seeded at a density of 400,000 cells/mL in 96-well black polystyrene plates or on round removable plastic supports in 24-well polystyrene plates (#174950 NUNC, Denmark). After 24 h, confluent HAEC were stained with 2.5 µM Orange Tracker (C34551, Thermofisher, Waltham, MA, USA) for 30 min at 37 °C. After a further 5 h, the cells were treated with TNF-α (50 ng/mL), and after another 20 h, PRP was added and the adhesion assay was performed. In the experiments for the evaluation of glucose effect, either 10, 20, 30 mM glucose was added to the culture 8 h after seeding and successively were treated as indicated above, for a total of glucose stimulation of 40 h.

### Platelet adhesion assay on HAEC under static conditions

PRP was diluted to a concentration of 150 × 10^9^/L in modified Tyrode’s solution (2 mM CaCl_2_, 1 mM MgCl_2_, 0.1% dextrose, 0.35% bovine serum albumin, 0.05 U/mL apyrase, pH 7.35)^24^ or in autologous PPP. Diluted PRP (100 µL/well) was then added to the HAEC and incubated for 30 min at 37 °C. Non-adherent platelets were removed by three gentle washes with Hank’s Buffer (HBSS). In some experiments, PRP was diluted in Tyrode’s solution to concentrations of 75, 150 and 300 × 10^9^/L to determine the optimal platelet concentration. In other experiments, platelets were pre-treated with tirofiban (0.3 and 3 µg/mL) for 3 min at 37 °C before exposure to HAEC or activated with TRAP (5 and 20 µM) directly in the plate. All experiments were performed in triplicate. Platelets adherent to HAEC were fixed with 4% paraformaldehyde (100 µL, Thermo Fisher Scientific, Waltham, MA, USA) for 30 min at RT, followed by three gentle washes with phosphate-buffered saline (1X PBS) and stained with FITC-conjugated anti-human CD61 (BD Biosciences, San Jose, CA, USA) diluted at a ratio of 1:50 in 1X PBS, followed by overnight incubation at 4 °C. The next day, platelets were visualized using an Axiovert A1 fluorescence microscope (Carl Zeiss, Oberkochen, Germany).

In additional experiments, HAEC were incubated overnight at 4 °C with ready-to-use FLEX monoclonal mouse anti-human CD31 (GA61061-2, DAKO Omnis, Glostrup, Denmark), washed twice with 1X PBS and then incubated with a secondary antibody (Alexa-Fluor 546 anti-mouse, IS3202, Immunological Sciences, Rome, Italy) diluted 1:1000 in 1X PBS for one hour at RT. Finally, the cells were washed twice with 1X PBS and incubated with Hoechst diluted 1:3000 in 1X PBS for nuclear visualization. Images were captured using an Axiovert A1 fluorescence microscope.

### Platelet adhesion assay on HAEC under high shear rate using a microfluidic device

Platelet adhesion experiments were also performed using a microfluidic device, as previously described^25^. The microfluidic devices consisted of six independent channels (1000 μm wide, 100 μm high and 3.2 cm long). The channel design followed previous recommendations, which suggested an aspect ratio of 10:1 (width: height) to minimize wall effects and ensure a homogeneous platelet distribution across the channel width. The microfluidic devices were fabricated using polydimethylsiloxane (PDMS, Sylgard 184, Dow Corning, Midland, MI, USA) and standard soft lithography techniques. The PDMS was prepared by mixing the pre-polymer with the curing agent in a 10:1 (w: w) ratio, degassed, poured over the master mold, and cured at 80 °C for 3 h. Fluidic inlet and outlet ports were created using a 1.5 mm diameter biopsy puncher. The PDMS chips were then permanently bonded to #0.6 microscope cover glasses via air plasma treatment.

The microfluidic channels were coated with 0.1% w/v gelatin and seeded with HAEC (90,000 −120,000 cells) to allow cell adhesion overnight at 37 °C, with 5% of CO_2_. After the medium change, 30 mM glucose was added to HAEC. Two hours later, the cells were stained with Orange Tracker (see HAEC culture) and after a further two hours, the medium was changed by adding TNF-α (40 ng/mL). On the following day, the medium was changed, and each channel was then washed with Hank’s balanced salt solution (HBSS) and checked for the absence of air bubbles. Hirudin-anticoagulated whole blood was perfused through the microchannels at 37 °C for 10 min at a shear rate of 950/sec. In some experiments, the whole blood was pre-incubated with the GP IIb/IIIa inhibitor (tirofiban, 3 µg/mL) for three minutes prior to perfusion. Following perfusion, the channels were washed with HBSS for 8 min and then fixed with 4% paraformaldehyde for 30 min at RT. The channels were then washed with 1X PBS, adherent platelets were labelled with FITC anti-human CD61 diluted 1:25 in 1X PBS and incubated overnight at 4 °C. The next day, adherent platelets were visualized using an Axiovert A1 fluorescence microscope.

### Image acquisition and analysis

Five images were acquired for each well and ten for each channel using 20x and 40x objective lenses, using MicroManager software. Platelet adhesion was then quantified using ImageJ 1.53k (NIH, USA) and a custom MATLAB script (R2022b, MathWorks, Natick, MA, USA). The script applies a threshold to distinguish between the dark background and brighter thrombi, assigning NaN values to background pixels. In this “masked” image, pixel clusters smaller than a single platelet’s diameter are removed. The processed image is then used to calculate platelet surface coverage, expressed as the percentage of pixels occupied by platelets. Results were saved in a.txt file for further analysis.

### Transmission electron microscopy (TEM)

To study the structural and ultrastructural features of platelets, samples were fixed in 2.5% glutaraldehyde in 0.13 M phosphate buffer pH 7.2–7.4 for 2 h, gently scraped from the flask, post-fixed in 1% osmium tetroxide, dehydrated using graded ethanol and propylene oxide, and embedded in epoxy resin. Several semi-thin sections of 1–2 μm thickness were prepared from the resin-embedded specimens and counterstained with toluidine blue for light microscopy. After preparation of the semi-thin sections, ultrathin sections of 50–70 nm thickness were prepared, counterstained with Pt-blue followed by lead citrate and examined with a Jeol JEM 1010 transmission electron microscope (Jeol, Tokyo, Japan).

### Scanning electron microscopy (SEM)

After performing the platelet adhesion assay, HAEC on coverslips were washed three times with HBSS and then fixed with 2.5% glutaraldehyde in 0.13 M phosphate buffer pH 7.2–7.4 for one hour at RT. The cells were then washed three times with 1X PBS, post-fixed with 1% osmium tetroxide for one hour at RT, and dehydrated through a series of ethanol concentrations ranging from 25% to 100%, and then with hexamethyldisilazane (HMDS) in proportion with ethanol 1:2, 1:1, 2:1, 100% HMDS. The samples were then left to evaporate slowly overnight. Coverslips containing dehydrated cells were mounted on standard SEM stubs and coated with a thin film of evaporated platinum, then placed in a Zeiss SIGMA (FE-SEM), operating at 5Kv at 15 mm of working distance. Images were analyzed using Esprit 1.8 software.

### Statistical analysis

Data from at least 4 experiments in triplicate in the static plate-based experiments are shown. In the microfluidics data from at least 4 experiments in single are shown. In the figures each point represents a different platelet donor. Data are shown as the mean ± standard deviation (SD). Differences between two groups were analysed using a paired t-test, while differences between multiple groups were analysed using a repeated measures one-way ANOVA followed by a Tukey’s post hoc test or a two-way ANOVA followed by a Bonferroni’s post hoc test, as appropriate. Statistical significance was defined as *p* < 0.05, and the analyses were performed using GraphPad Prism, version 9.1.5 (GraphPad Software Inc., San Diego, CA, USA).

### Data Availability

The data supporting the findings of this study are included in this published article. Raw data generated and/or analysed during the current study are available from the corresponding author, upon reasonable request.

## Supplementary Information

Below is the link to the electronic supplementary material.

Supplementary Material 1
