# Supplementary material for: High glucose enhances inflammation-driven platelet adhesion to endothelial cells in vitro
Source: Sci Rep. 2025 Dec 1;16:127. doi: 10.1038/s41598-025-28746-4 (PMC12764815; doi:10.1038/s41598-025-28746-4)
Supplement: Supplementary file 1 — Supplementary Material 1 [file 41598_2025_28746_MOESM1_ESM.docx]

**Supplementary data**

**Figure legend**

**Figure suppl 1. Experimental conditions for adhesion assay: determination of platelet and TNF-α concentration.**


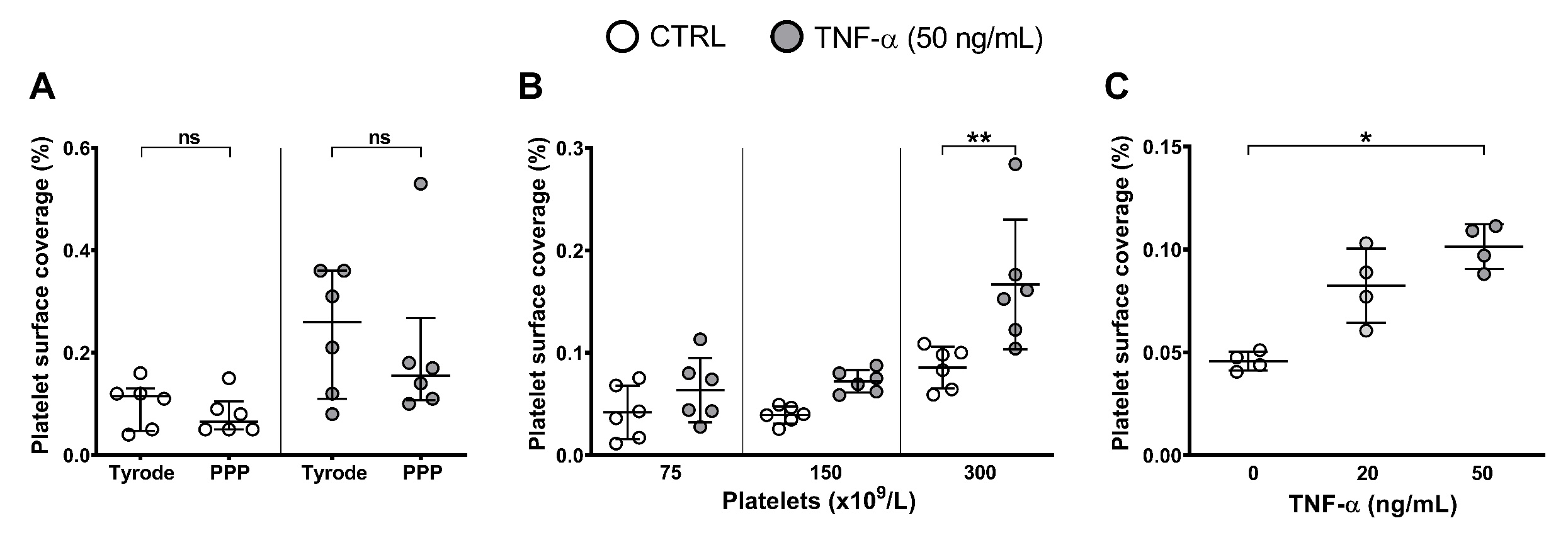


Platelet adhesion was evaluated by platelet surface coverage in control and TNF-α-treated HAEC. (**A**) Comparison between Tyrode’s solution and autologous PPP as dilution buffers for PRP revealed no significant differences in platelet adhesion. (**B**) Variation of platelet adhesion to untreated and TNF-α (50 ng/mL)-treated HAEC is dependent on platelet concentration. (**C**) Platelet adhesion increases with higher TNF-α concentrations. Data are shown as mean percentage of adhering platelets ± SD from 4 different experiments. RM one-way ANOVA followed by Tukey’s *post hoc* test (**p<0.01).

**Figure suppl 2. Platelet aggregation measured by light transmission aggregometry (LTA).**


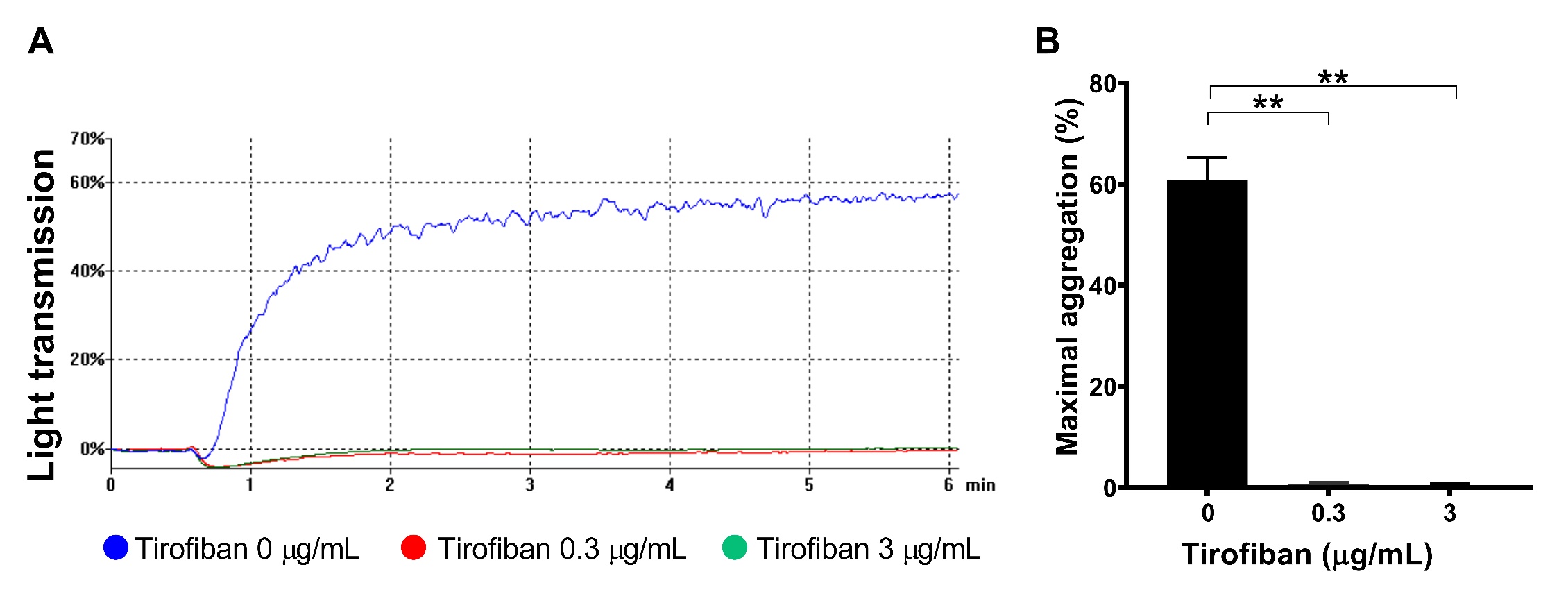


(**A**) Representative traces of platelet aggregation induced by TRAP (20 µM) in tirofiban (3 µg/mL, green line and 0.3 µg/mL, blue line)-treated platelets. (**B**) Maximal platelet aggregation (%) of hirudin-anticoagulated PRP following stimulation with 20 µM TRAP in presence of absence of tirofiban (0.3 and 3 µg/mL). Data are shown as the mean percentage of maximal aggregation ± SD (paired t test) (**p<0.01).

**Figure suppl 3. Platelet activation with TRAP does not increase adhesion to TNF-α-treated HAEC.**

0
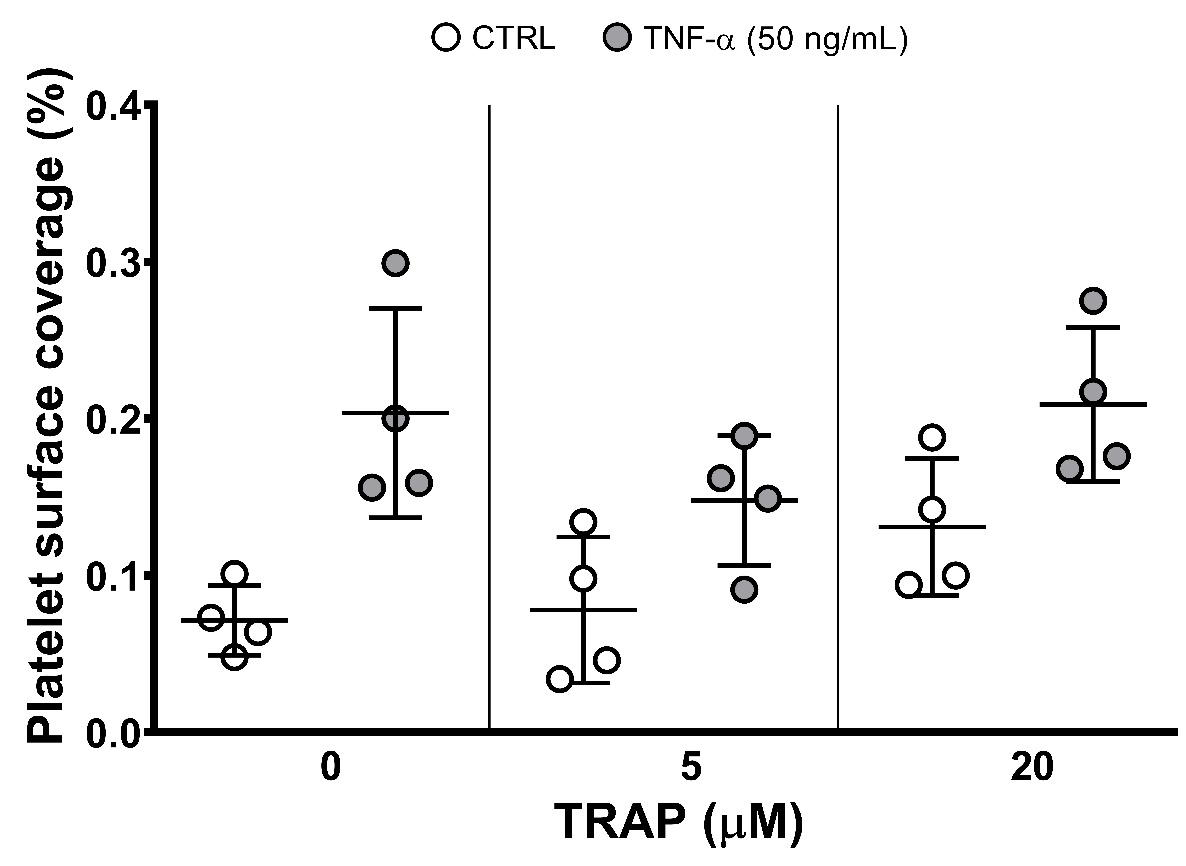


Platelet adhesion was evaluated in HAEC treated with and without TNF-α (50 ng/mL), utilizing PRP after activation with two different concentrations of TRAP (5 and 20 µM). The mean percentage of adhering platelets ± SD from 4 different experiments is shown. RM two-way ANOVA followed by Bonferroni’s *post hoc* test.

**Figure suppl 4. Flow diagram illustrate the two-phase experimental pipeline for studying platelet–endothelium interactions.**


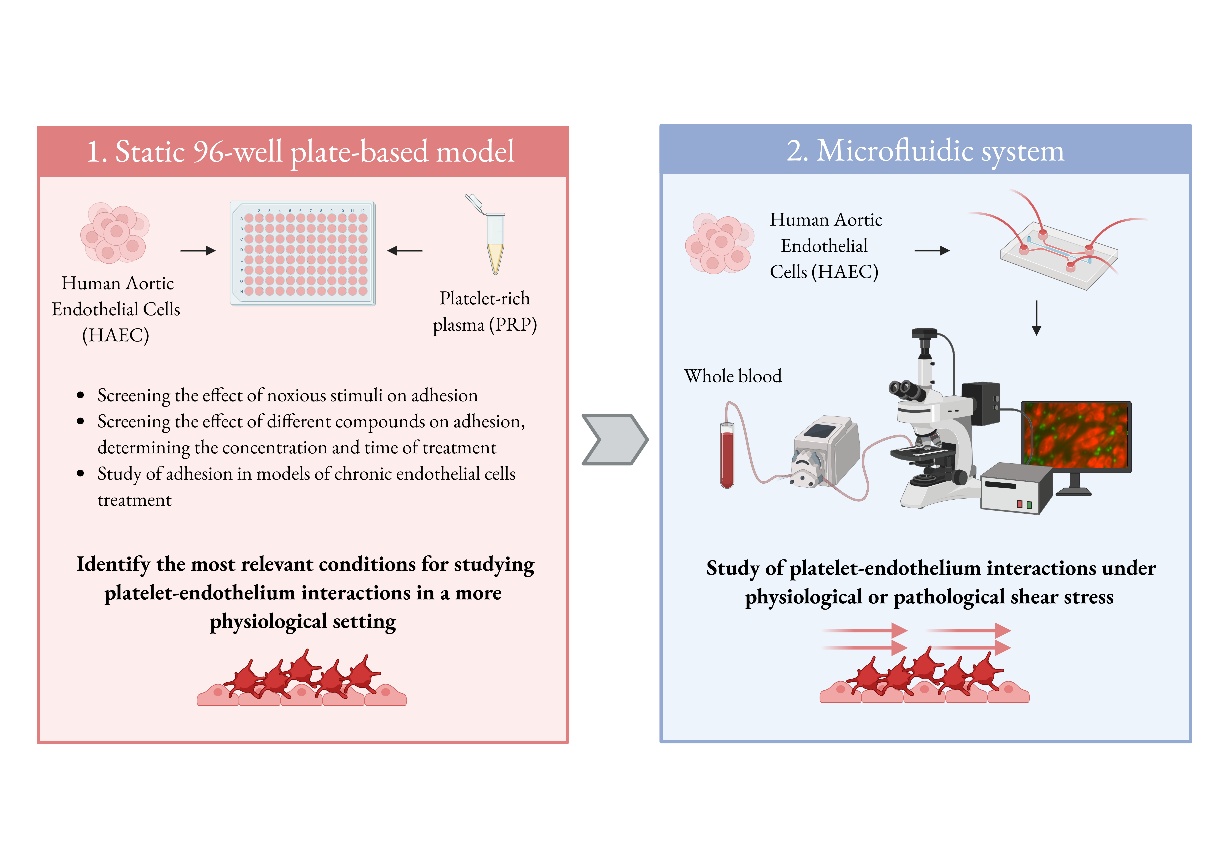


The simple 96-well plate-based model is first used to identify and optimize experimental conditions, providing essential preliminary data. Once the most relevant conditions are identified, they are then tested to study platelet–endothelium interactions under more physiologically relevant dynamic flow conditions.
